# The menstrual cycle: an overlooked vital sign in psychiatry?

**DOI:** 10.1136/bmjment-2024-301463

**Published:** 2025-03-03

**Authors:** Katie FM Marwick, Thomas J Reilly, Stephanie Allan, Ellen Golightly

**Affiliations:** 1 Division of Psychiatry, The University of Edinburgh, Edinburgh, UK; 2 Department of Psychiatry, University of Oxford, Oxford, UK; 3 School of Health and Wellbeing, University of Glasgow, Glasgow, UK; 4 Chalmers Centre for Sexual and Reproductive Health, NHS Lothian, Edinburgh, UK

**Keywords:** Adult psychiatry, Depression & mood disorders, Schizophrenia & psychotic disorders, Suicide & self-harm

## Abstract

We argue that psychiatry has overlooked a significant modifiable risk factor for mental illness in female patients: cyclical symptoms associated with the menstrual cycle. Premenstrual dysphoric disorder has recently been included in the International Classification of Diseases-11, while there is growing evidence that menstrual cycle stage can impact mental health symptoms transdiagnostically and influence important outcomes such as suicide or admission to hospital. Intervention is possible using existing widely available hormonal treatments. Asking patients about the relationship between their menstrual cycle and mental health is an opportunity to improve diagnosis and treatment that should no longer be neglected.

## The menstrual cycle: an overlooked vital sign in psychiatry?

 Should the menstrual cycle phase be considered a ‘vital sign’ in psychiatry, as recommended for some other areas of medicine^1^? We are living through a ‘menstrual moment’ when the long-neglected intersection of female’s reproductive and mental health is now becoming an acceptable part of public discourse. This is a valuable opportunity to improve patient care. Here we summarise the associations between the menstrual cycle and psychiatric symptoms and highlight opportunities to harness this potentially modifiable risk factor in clinical practice. We share the lived experience of our author, SA, as a real-world example. Our key message is that we should be asking patients more about how their menstrual cycle affects their mental health and using this information to inform diagnosis and management.

### Premenstrual dysphoric disorder (PMDD) is now an official diagnosis

PMDD is the archetypal menstrual cycle mood disorder that comprises affective, cognitive and somatic symptoms confined to the premenstrual phase of the cycle associated with significant distress or functional impairment. PMDD is a new diagnosis in the genitourinary chapter of the 11th revision of the International Classification of Diseases (ICD-11). Although official recognition has been recent, this is the culmination of decades of research. Diagnosis can be reliably made using prospective daily symptom rating scales over two menstrual cycles. In large epidemiological studies, the point prevalence has been estimated at 1.6% when strictly adhering to diagnostic criteria.^2^

PMDD is thought to be caused by an altered sensitivity to normal cyclical changes in oestradiol and progesterone. Symptoms remit with either suppression of ovarian hormones or high steady levels.^3^ To this end, the Royal College of Obstetricians and Gynaecologists recommends combined oral contraceptives be considered as a first-line pharmacological treatment for PMDD,^4^ supported by a network meta-analysis.^5^ What is less widely known is that the guidelines also recommend suppressing ovulation for ‘premenstrual exacerbation of an underlying disorder’, such as depression.^4^ The other first-line option for PMDD, selective serotonin reuptake inhibitors (SSRIs), have a much faster onset of action in PMDD (within 48 hours) than in typical depression.^6^ Another difference in PMDD is that SSRIs are equally effective when given only during the premenstrual phase compared with continuous dosing.^7^ As with any medication, hormonal contraceptives and SSRIs have side effects and the decision to prescribe is a patient-centred risk–benefit discussion.

### Perimenstrual exacerbation is a transdiagnostic risk factor in mental health

**Figure 1:**
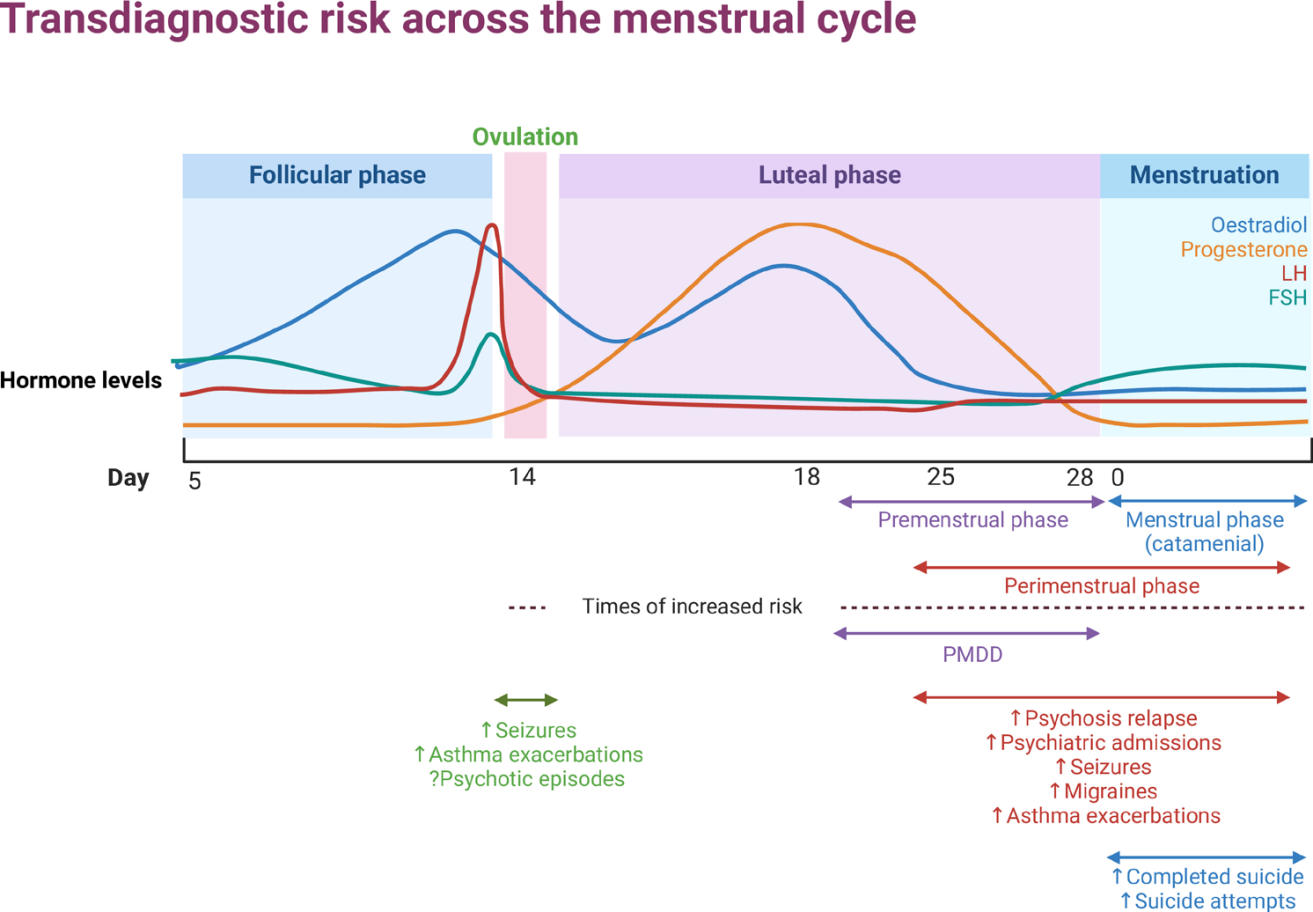
Schematic representation of hormone fluctuations during the menstrual cycle with associated risk for various disorders. The list of disorders provided is indicative and not comprehensive. The cycle is standardised to 28 days with ovulation at 14 days; in practice, this shows marked interindividual and intraindividual variation. Cycle phase definitions can vary between studies; here the premenstrual phase is day 18–day 28, menstrual (catamenial) phase day 1–day 5, perimenstrual phase day 24–day 5. FSH, follicle stimulating hormone; LH, luteinising hormone; PMDD, premenstrual dysphoric disorder. Created with BioRender.com.

PMDD symptoms resolve completely in the follicular phase. However, a wide range of psychiatric disorders have evidence of perimenstrual exacerbation: symptom worsening during the days before and during menstruation, without complete resolution of symptoms during the follicular phase. Premenstrual symptom worsening has been found in prospective studies of psychotic disorders, bipolar disorder, depression, panic disorders, eating disorders and emotionally unstable personality disorder.^8^ Alcohol and nicotine consumption also increase perimenstrually.^9^ Perimenstrual exacerbation is not limited to mental disorders: physical health conditions such as epilepsy,^10^ migraine^11^ and asthma^12^ have all shown worsening during this phase of the cycle. Severe mental health outcomes are also associated with cycle phase: meta-analyses have found that the perimenstrual phase has an increased risk of psychiatric admission for all diagnoses,^13^ particularly psychosis,^14^ and menstruation is associated with an increased rate of suicide attempts and completed suicide^13^ (figure 1).

SA describes how she experiences menstrual cycle changes in her psychotic illness:

I think I always had negative emotional changes before my periods, though I did not realise it as it was ‘my normal’. When I was diagnosed with psychosis and then schizophrenia, this continued. I am pretty sure the three times I was hospitalised were before a period started. I know my period is coming as I get early morning awakenings; this lack of sleep is then associated with what might be called ‘basic symptoms’ where I can see lines in the pavement move and the world seems a bit brighter and louder. I also get irritable around this time and can get more ‘stuck’ on thinking patterns. I feel my brain is connected in a different way with more ‘aha!’ moments when I see connections which in retrospect are not there.

### An opportunity for stratified medicine

If a patient experiences mental state changes during the menstrual cycle, are they at risk of developing mental illness at other times of reproductive transition, such as childbirth and the perimenopause? Preliminary evidence suggests this is the case. Premenstrual syndrome is the strongest risk factor for postpartum depression identified by meta-analysis.^15^ A large survey of over 500 women with clinician-assessed major depressive disorder found that having premenstrual mood symptoms predicted both postpartum and perimenopausal mood symptoms.^16^ Improving understanding of ‘Hormonal trigger events’ has been identified as a research priority in PMDD by a recent UK-wide consultation.^17^

### An opportunity for intervention

What can be done if a patient reports menstrual cycle-related symptom worsening? At the simplest level, routine enquiry of menstrual cycle phase can improve understanding of triggers by uncovering a pattern of illness that is not always apparent to the patient. SA comments:

I was glad when my CPN first pointed out the link between my periods and psychotic symptoms as it wasn’t obvious to me.

Further, there is growing evidence that hormonal approaches can be efficacious for severe mental illness. A meta-analysis of six randomised controlled trials using adjunctive oestradiol in women with schizophrenia found benefits over placebo in both positive and negative symptom scales^18^ and receiving hormone replacement therapy reduced hospitalisation rate for schizophrenia by 16%.^19^ If a female patient in mid-life is not responding to standard treatment for psychosis, evidence suggests adjunctive hormonal treatment should be considered.

### An opportunity to enhance dignity

Respectfully enquiring about menstrual cycle phase may also be a way to support inpatients experiencing menstruation in as dignified and humane a way as possible. A 2024 patient-led report^20^ found that patients’ needs while menstruating in hospital are often overlooked, with inadequate access to period products, inadequate means of disposing of products and restriction of usual self-care approaches such as analgesia. SA comments:

The nurses thought they were helping my mental health by insisting I got out of bed but when I’m on my period I just want to rest.

For some patients, the intimate and gendered nature of menstruation means that negative staff interactions in this regard resonate with their experience of gendered violence and trauma. This is another blind spot: surveys showed that staff consistently think they support patients’ menstrual needs better than patients feel they are supported.^20^

## Barriers

There are many unanswered questions in this area. Why has psychiatry not yet embraced the opportunity provided by enquiry about the menstrual cycle box 1? Why are cognitive changes associated with the menstrual cycle in the genitourinary chapter of ICD-11, whereas cognitive changes associated with all other physical states (neurocognitive disorders) are in the mental, behavioural or neurodevelopmental disorder chapter? Is this a consequence of hundreds of years of male-centred research and practice in Western medicine? Is ascribing changes in mental state to changes in hormones seen as reductive or invalidating? Do psychiatrists feel unfamiliar or intrusive asking female patients about menstruation? S.A comments:

Being asked about periods is no more intrusive than being asked about suicidal thoughts. It’s just a bodily function, like sleep.

Box 1 **Clinical utility of asking females presenting with mental health symptoms about their menstrual cycle**

**Diagnosis**

- May have a primary diagnosis of premenstrual dysphoric disorder.
- May experience premenstrual exacerbation of an underlying mental disorder.
- May have distressing or disruptive physical symptoms related to menstruation or menopause which are influencing mental health.

**Management**

- Understanding why symptoms arise can reduce self-blame.
- May benefit from acknowledgement of psychosocial challenges and stigma associated with menstruation and menopause.
- May need practical support with access to period products, particularly if living in poverty or admitted to hospital.
- May benefit from advice on lifestyle optimisation for premenstrual or perimenopausal symptoms.
- Menstrual cycle stage could be incorporated into suicide prevention safety plans and relapse prevention plans.
- May benefit from treatments to reduce ovarian hormone fluctuation.
- May need further investigation or treatment of abnormal uterine bleeding.

**Prognosis**

- May be planning a pregnancy and worried about the effect on mental health.
- May be approaching the menopause and worried about effect on mental health.

Our own clinical experience and patient advisory panel input suggests that asking patients about menstruation is welcomed. People with lived experience tell us that they are noticing patterns in their symptoms and have questions about what hormonal treatments are best and what to expect during pregnancy and menopause. Improving the evidence base will allow us to better support our patients in informed, shared decision-making.

There is little coverage of menstrual cycle effects on mental health in undergraduate or postgraduate medical or psychiatric curricula, or in the exam syllabus for membership of the Royal College of Psychiatrists (RCPsych). This is a disservice to female patients, representing a major blind spot in psychiatry and contributing to the invisibility and hesitancy around female-specific disorders like PMDD. To recognise the potential contribution of the cycle and to tailor treatments for individual patients, we need to equip the next generation of psychiatrists with knowledge and awareness. A positive step is the first RCPsych Continuing Professional Development learning module on PMDD, published in 2023.

## Conclusion

A systemic change in the assessment and care of female mental health is overdue. The effects of the menstrual cycle on mental health are too important to ignore. The first step is simply to routinely enquire about menstrual cycle effects. Doing so can provide valuable information about diagnosis, relapse triggers, prognosis and treatment options. Importantly, asking about menstrual cycle stage enhances, rather than diminishes, patients’ dignity, and acknowledges and normalises a topic patients and carers want to know about. Mental health professionals and our patients stand to benefit from harnessing knowledge about how the menstrual cycle affects mental health: this is a call to action to bring the menstrual cycle into mental health training, research and practice.
